# Experimental evidence for species-dependent responses in leaf shape to temperature: Implications for paleoclimate inference

**DOI:** 10.1371/journal.pone.0218884

**Published:** 2019-06-21

**Authors:** Melissa L. McKee, Dana L. Royer, Helen M. Poulos

**Affiliations:** 1 Department of Earth and Environmental Sciences, Wesleyan University, Middletown, Connecticut, United States of America; 2 College of the Environment, Wesleyan University, Middletown, Connecticut, United States of America; Baylor University, UNITED STATES

## Abstract

In many woody dicot plant species, colder temperatures correlate with a greater degree of leaf dissection and with larger and more abundant leaf teeth (the serrated edges along margins). The measurement of site-mean characteristics of leaf size and shape (physiognomy), including leaf dissection and tooth morphology, has been an important paleoclimate tool for over a century. These physiognomic-based climate proxies require that all woody dicot plants at a site, regardless of species, change their leaf shape rapidly and predictably in response to temperature. Here we experimentally test these assumptions by growing five woody species in growth cabinets under two temperatures (17 and 25°C). In keeping with global site-based patterns, plants tend to develop more dissected leaves with more abundant and larger leaf teeth in the cool treatment. Overall, this upholds the assumption that leaf shape responds in a particular direction to temperature change. The assumption that leaf shape variables respond to temperature in the same way regardless of species did not hold because the responses varied by species. Leaf physiognomic models for inferring paleoclimate should take into account these species-specific responses.

## Introduction

In most regions of the world, the proportion of toothed woody dicot species, as well as species-mean leaf tooth size, tooth number, and leaf dissection, inversely correlate with mean annual temperature (MAT) [1–10]. In short, leaves in cold climates are more likely to have toothed leaf margins with larger and more numerous teeth. Many leaf-functional traits, including size and shape (physiognomy), can be measured in leaf fossils. As such, the paleobotanical community has developed both univariate and multivariate approaches, or leaf-physiognomy climate-models, to quantitatively reconstruct aspects of paleoclimate from leaf fossils [3–10].

Royer and Wilf [11] measured photosynthesis and transpiration in toothed and untoothed leaf margins to investigate the biological underpinnings between leaf teeth and MAT. They found that toothed species often had higher gas-exchange rates in their teeth than in their leaf interior during the first few weeks of the growing season. The corresponding increase in sap flow in young, expanding leaves should increase the delivery of nutrients to the entire lamina. This function of leaf teeth could be increasingly adaptive in colder climates (with corresponding shorter growing seasons) because leaf teeth could help plants ramp up to maximum carbon production rates sooner relative to an equivalent leaf without teeth. In warmer climates with longer growing seasons, the water cost associated with teeth could outweigh any benefits for maximizing the growing season length [11]. Alternative functional explanations for leaf teeth include the release of excess root pressure [12], the mechanical support associated with leaf thickness [13], and the pattern and duration in which leaf primordia are packed into resting buds [14].

All current leaf-physiognomy climate-models are site-based [3–10]: that is, climate is estimated from the across-species mean of leaf physiognomy. A tacit assumption with these models is that site-mean leaf shape responds rapidly (tens-to-hundreds-of-years) and predictably to climate change. By growing identical seed lines of red maple (*Acer rubrum* L.) in growth cabinets, Royer [15] found that leaves that developed under cooler temperatures were more dissected and had more teeth. These experiments provide strong evidence for the direct and very rapid effect of growth temperature on the morphology of leaf teeth. These experiments also show a response in leaf shape within a single species—and within a single generation—that is consistent with global site-based patterns. Chitwood et al. [16] found similar patterns in field-grown *Vitis* within the span of just two growing seasons, where one year was colder and dryer than the other.

How applicable are plastic responses in a single species to site-based methods, especially considering that: 1) fossil deposits rarely resolve timescales relevant for plastic responses; 2) in present-day compilations of natural populations across the globe, the primary reason why site-mean leaf physiognomy covaries with climate is because species composition differs across sites [1–10], and in cases where physiognomy in natural populations covaries with climate within a single species (e.g., *A*. *rubrum* [17]), common-garden experiments suggest that differences in genotype are driving at least some of the differences in physiognomic expression [18]; and 3) leaf physiognomy within species does not always respond to climate, either plastically or through changes in genotype [17], [19].

We see at least two reasons for why documenting plastic responses can be relevant for leaf-physiognomy climate-models. First, growth-cabinet experiments provide strong evidence for a causal link between leaf physiognomy and climate because—in contrast to observations in natural populations—other environmental factors can be controlled for. Establishing a causal link boosts confidence that these models can be applied generally, including to fossil settings. Second, if leaf physiognomy can respond to changes in climate within the lifetime of an individual, this increases confidence that fossil assemblages (site means) can faithfully record climate, even during periods of rapid climate change, and not a preceding climate state.

With these motivating principles in mind, how general are the plastic responses exhibited by *A*. *rubrum*? Here we test how the leaf physiognomy in five dicot tree species responds when grown in growth cabinets of contrasting temperature. If the plasticity observed in *A*. *rubrum* is common in other taxa, then this would increase the likelihood that reconstructions of climate from the site-mean of fossil leaf physiognomy are robust, even during times of rapid climate change.

We evaluated three key questions: 1) Are leaves grown in cooler temperatures “toothier” and more dissected? This is arguably the most important question for paleobotanists aiming to reconstruct climate from fossil leaf physiognomy. 2) How quickly can leaf physiognomy respond to temperature change? We grew four species from seed and three from saplings (two of the species were grown from both seed and saplings). Because the saplings had previously experienced a different environment, any physiognomic response to temperature in the growth cabinets would underscore the speed at which these changes happen, even within a single growing season. 3) Do all species respond in the same way? Because current leaf-physiognomy climate-models are site-based, they require an assumption, on some level, that leaf physiognomy responds to climate in the same way, regardless of the species [5], [20–22]; or, more directly, that the across-species mean of leaf physiognomy always responds to climate in the same way. Our multi-species study design allows us to partly address this question.

## Materials and methods

We grew four species from seed (*Acer negundo* L., *Betula lenta* L., *Carpinus caroliniana* Walter, and *Quercus rubra* L.) and three species from transplanted saplings (*Acer negundo*, *Carpinus caroliniana*, and *Ostrya virginiana* (Mill.) K.Koch) (see S1 Text for details). We selected these species because each has natural populations along the east coast of the United States with leaf shapes that tend to covary with climate in a manner that mirrors site-mean patterns [19].

Seeds and saplings were randomly divided into two temperature treatments. Each group was grown in an independently controlled growth cabinet (Conviron E7/2; Winnipeg, Manitoba, Canada). Both cabinets were set to a 17-hour photoperiod with a 30-minute simulated dawn and dusk, and a 500 ppm CO_2_ concentration (actual = 503 ± 10.4 ppm [1σ]). The warm treatment had a target day/night temperature of 28/19°C (actual = 27.9 ± 0.5°C and 19.2 ± 0.7°C), with a time-weighted mean temperature of 25.0°C. The cool treatment had a target day/night temperature of 20/11°C (actual = 20.0 ± 0.3°C and 11.3 ± 1.1°C), with a time-weighted mean temperature of 17.1°C. The cabinets differed somewhat in relative humidity (76.5 ± 1.8% and 90.0 ± 3.6%); because of this, the warm and cool treatments were alternated between cabinets every two weeks to minimize cabinet effects.

Leaves were harvested after three months. From each plant, we selected five young, fully expanded leaves that developed from buds that formed while the plant was in the growth cabinet. In most cases, seven plants per species × temperature were analyzed (exceptions: ten for *B*. *lenta* and five for *O*. *virginiana*). Leaves were photographed immediately (Nikon D5300 camera, Nikon, Melville, New York, USA). The procedure for measuring leaf physiognomy is discussed fully by Royer et al. [19] and Huff et al. [23]. Briefly, in Photoshop (Adobe Systems, San Jose, California, USA), minor defects along the leaf margin were corrected using the line tool and petioles were separated from the leaf blade. Teeth were then digitally separated from the leaf blade; most teeth are bounded by two sinuses, but see Royer et al. [19] and Huff et al. [23] for exceptions. Leaf physiognomy was then quantified with ImageJ software (http://rsbweb.nih.gov/ij/). In addition, we measured the fractal dimension of leaves (see S1 Text for details). All measured variables are related to tooth abundance, tooth size, or degree of leaf dissection; see Table 1 for a complete list, along with definitions. All data are presented in S1 Dataset. Tooth size in *Q*. *rubra* was highly variable, including many instances of lobes (following Royer et al. [19]), leading to difficult-to-interpret patterns; because of this, we exclude tooth-size variables for *Q*. *rubra*.

**Table 1:** Physiognomic variables and definitions.

Variable	Definition
**Tooth abundance**	
Number of teeth	Total number of primary and secondary^a^ teeth
Number of teeth / internal perimeter	Total number of teeth / internal perimeter (perimeter of leaf after leaf teeth are removed) (cm-1)
Number of teeth / blade area	Total number of teeth / blade area (cm-2)
**Tooth size**	
Tooth area	Total area of teeth (cm2)
Average tooth area	Tooth area / number of primary teeth (cm2)
Tooth area / internal perimeter	Tooth area / internal perimeter (cm)
Tooth area / blade area	Tooth area / blade area (dimensionless)
**Leaf dissection**	
Circularity	4π × [leaf area / (leaf perimeter)2] (dimensionless)
Perimeter ratio	Leaf perimeter / leaf internal perimeter (dimensionless)
Feret diameter ratio	Feret diameter (diameter of a circle with the same area as the leaf) / major length (longest measured line across the leaf blade) (dimensionless)
Fractal dimension	Degree of an object’s boundary fragmentation over multiple scales (dimensionless)

Global patterns [6] predict a negative correlation with mean annual temperature for all variables except circularity.

^a^ Secondary teeth are teeth that are located on larger, primary teeth [19].

**Fig 1:**
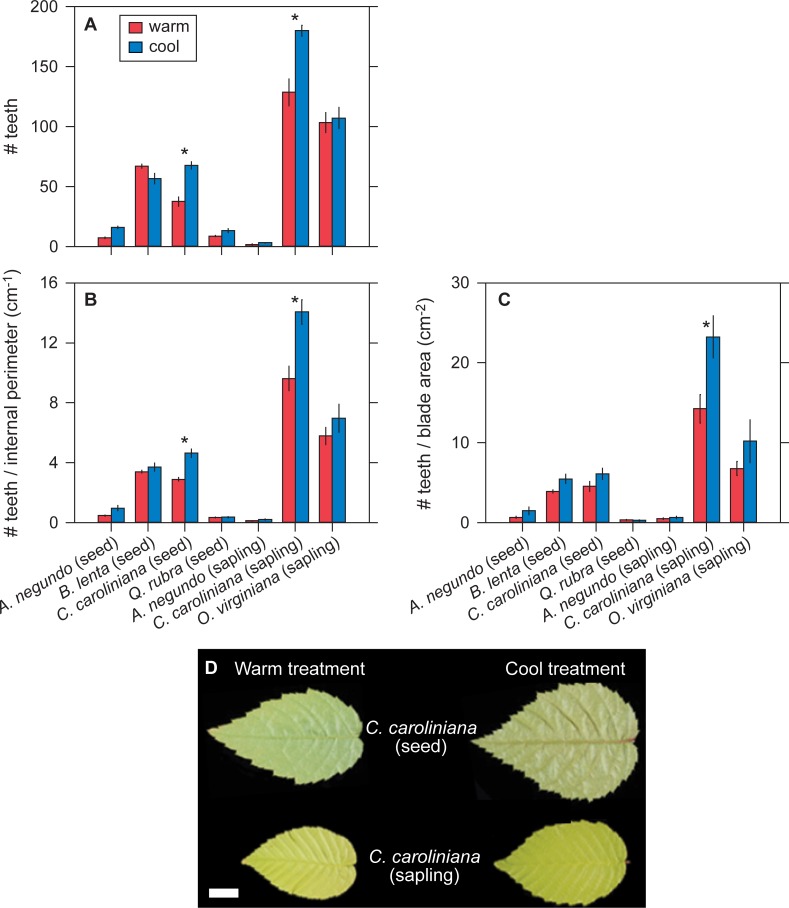
Sensitivity of tooth abundance variables to temperature. (A-C) Comparisons of the species grown in the growth cabinet experiment for tooth abundance variables. Values are means ± 1 s.e.m. * indicates a significant difference (*P* < 0.05) based on estimated marginal means (see Materials and Methods). Global site-based patterns [6] would predict higher values in the cool treatment for all variables. (D) Representative leaves from species in this experiment that show significant differences in tooth abundance between the two treatments. Scale bar = 1 cm.

**Fig 2:**
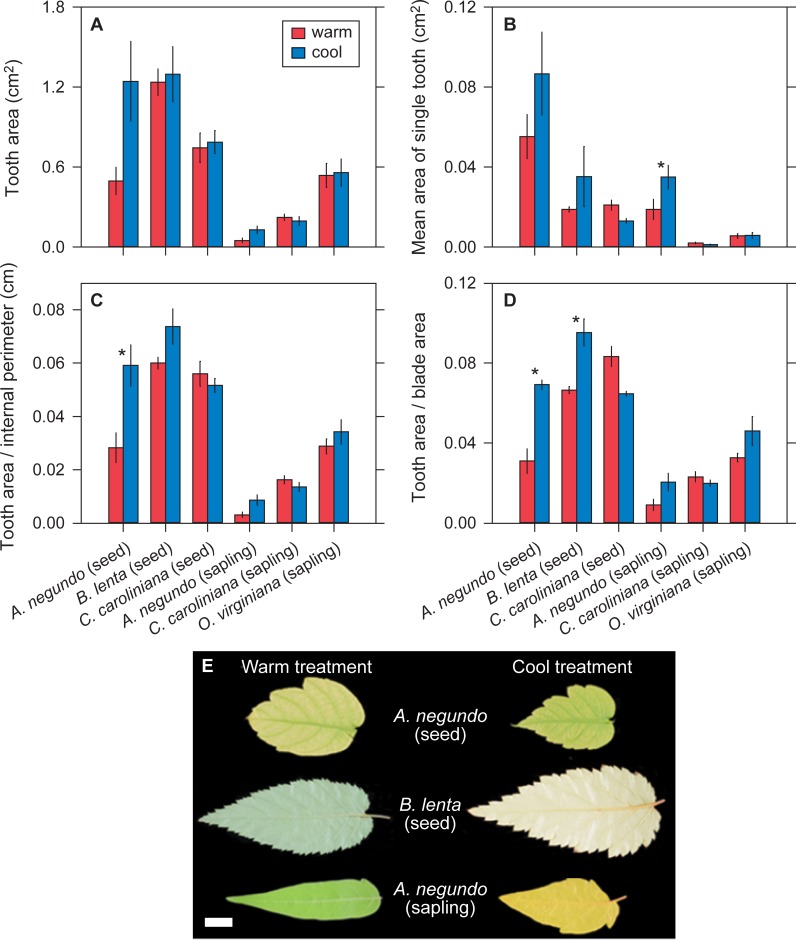
Sensitivity of tooth size variables to temperature. (A-D) Comparisons of the species grown in the growth cabinet experiment for tooth size variables. Values are means ± 1 s.e.m. * indicates a significant difference (*P* < 0.05) based on estimated marginal means (see Materials and Methods). Global site-based patterns [6] would predict higher values in the cool treatment for all variables. (E) Representative leaves from species in this experiment that show significant differences in tooth size between the two treatments. Scale bar = 1 cm.

**Fig 3:**
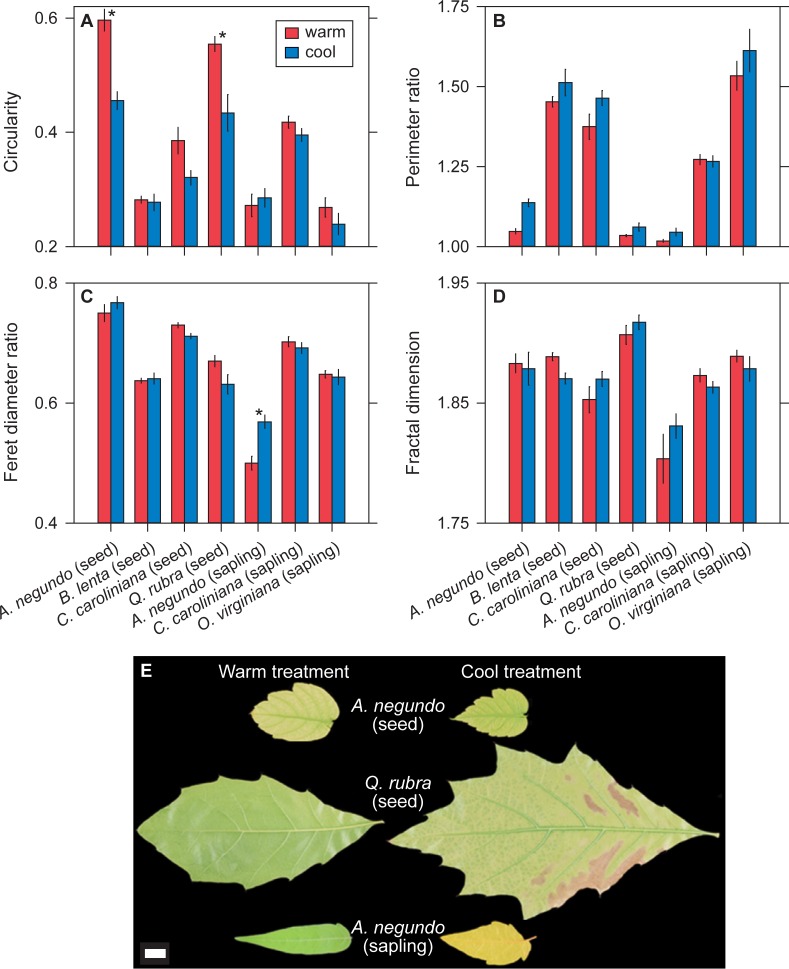
Sensitivity of leaf dissection variables to temperature. (A-D) Comparisons of the species grown in the growth cabinet experiment for leaf dissection variables. Values are means ± 1 s.e.m. * indicates a significant difference (*P* < 0.05) based on estimated marginal means (see Materials and Methods). Global site-based patterns [6] would predict higher values in the cool treatment for perimeter ratio, feret diameter ratio, and fractal dimension; and lower values for circularity. (E) Representative leaves from species in this experiment that show significant differences in leaf dissection between the two treatments. Scale bar = 1 cm.

The unit of replication for all statistical tests was the number of plants per species × temperature treatment. For most analyses, we first divided the data into plants grown from seed and saplings. We did this because the physiognomy for the two species grown from both seed and saplings was often quite different (*A*. *negundo* and *C*. *caroliniana*; e.g., Figs 1D, 2E and 3E; see also S5 Table). We tested for differences in each physiognomic variable between warm and cool treatments with linear models using the lm function in R [24]. First, we evaluated species-level differences in leaf physiognomy with temperature via estimated marginal means in the emmeans package in R [25], with a Dunn-Šidák correction to account for multiple comparisons. Second, we compared leaf physiognomy across species, with species, growth temperature, and the species × temperature interaction as the fixed effects. To test the significance of temperature across species, we computed an ANOVA within the linear model using the anova function in R. An advantage of pooling species is that the statistical power is improved for discerning small but pervasive differences that may not be statistically significant within individual species. Third, to test if the physiognomic responses to temperature were dependent on life stage, we constructed a linear model based on the two species grown from both seed and saplings, with life stage, temperature, and the life-stage × temperature interaction as the fixed effects. For all tests, *P* < 0.05 was considered statistically significant.

## Results

The directionality of leaf physiognomic responses to temperature are generally consistent with global site-based patterns: leaves from the cool treatment tend to have more numerous teeth, larger teeth, and be more dissected (lower circularity and higher perimeter ratio) (Figs 1–3). Two leaf dissection variables—feret diameter ratio and fractal dimension—show a more mixed response. At the level of individual species, though, very few of these temperature comparisons are significant (asterisks in Figs 1–3; see also S1 & S2 Table).

When we pool species, we find that the differences in leaf physiognomy between temperature treatments are often significantly different. For species grown from seed, all three tooth abundance variables (number of teeth [*P* = 0.002], ratio of number of teeth to internal perimeter [*P* < 0.001], ratio of number of teeth to blade area [*P* = 0.002]), two of the tooth size variables (ratio of tooth area to internal perimeter [*P* = 0.003] and ratio of tooth area to blade area [*P* < 0.001]), and two of the leaf dissection variables (circularity [*P* < 0.001] and perimeter ratio [*P* < 0.001]) are all significantly greater in the cool treatment, which is in keeping with global site-based patterns (S3 Table). Six out of the eleven variables have significant interaction terms in the effect of temperature among species, meaning that the physiognomic responses to temperature are often species-specific (S3 Table).

The saplings show similar but fewer significant patterns: consistent with global site-based patterns, all three tooth abundance variables (number of teeth [*P* < 0.001], ratio of number of teeth to internal perimeter [*P* < 0.001], ratio of number of teeth to blade area [*P* = 0.005]), one tooth size variable (ratio of tooth area to blade area [*P* = 0.04]), and one leaf dissection variable (feret diameter ratio [*P* = 0.02]) are significantly greater in the cool treatment. Five variables have a significant temperature × species interaction (S4 Table).

For the two species that were grown both from seed and saplings (*A*. *negundo* and *C*. *caroliniana*), only two physiognomic variables have a significant temperature × life-stage interaction: tooth area (*P* = 0.05) and circularity (*P* = 0.05) (S5 Table). This means that for these two variables, temperature does not have the same effect on plants grown from seed vs. saplings.

## Discussion

For plants grown from seed, seven out of the eleven measured leaf physiognomic variables respond significantly to temperature. In all cases, physiognomy varies in the same direction predicted from global site-based patterns, with leaves from the cool treatment tending to be more dissected and to have larger and more numerous teeth. These results are consistent with the previous experiments with *A*. *rubrum* [15], [18]: leaf physiognomy in deciduous, toothed tree species can respond quickly to temperature and in the direction predicted from global site-based patterns. These results are encouraging for those who wish to use leaf physiognomic variables for paleoclimatic reconstructions using leaf fossil data.

For plants grown from saplings, five physiognomic variables respond significantly to temperature. Similar to the plants grown from seed, all three tooth-abundance variables respond, but the sapling responses are comparatively weaker for the tooth area and leaf dissection variables. This may mean that saplings transplanted to a new environment are not as responsive to temperature as plants grown from seed in a single environment. But the difference in our experiment is not large (seven vs. five significant variables); moreover, for the two species grown both from seed and saplings, only two physiognomic variables have a significant temperature × life-stage interaction. Longer-term experiments with more than two species are needed to test more fully how quickly established plants can alter their leaf physiognomy to changes in temperature. This issue is important for fossil studies because if leaf teeth cannot respond quickly enough to rapid changes in their environment, fossil leaves may reflect the environmental conditions of a time prior to their deposition.

A significant temperature × species interaction is present for most of the physiognomic variables that respond significantly to temperature. This means that the response of leaf physiognomy to temperature can be species-specific. All current leaf-physiognomy climate-models are site-based [3–10], where different sets of species are compared across sites. If species-specific responses in leaf physiognomy to temperature change are common, then this raises the concern that the site-based physiognomy-climate relationships that we observe today may change as temperature changes. Multiple studies, based on species-rich data sets from North America, South America, and Asia, identify the presence of a species-specific signal in many of the correlations between climate and tooth size, tooth number, and leaf dissection [5], [20–22]. Our results support the more general call to incorporate phylogenetic signal into leaf-physiognomy climate-models [22].

## Conclusions

Our experiments support the view that leaf shape generally changes predictably and rapidly in response to temperature change. Critically, the temperature effects on leaf shape are usually species-specific. Because of this, we recommend that leaf-physiognomy climate-models for reconstructing paleotemperature from fossil leaves take into account these species-specific responses.

## Supporting information

S1 Text Supplementary methods. Methods for cultivating plants in the growth cabinet experiment and for fractal dimension measurements.
(PDF)

S1 Table Linear model results for seedlings testing for the effect of temperature on leaf shape within species. (PDF)

S2 Table Linear model results for saplings testing for the effect of temperature on leaf shape within species. (PDF)

S3 Table Analysis of variance of a linear model testing for differences in leaf shape in seedlings across species, temperature (cool or warm), and the species × temperature interaction. (PDF)

S4 Table Analysis of variance of a linear model testing for differences in leaf shape in saplings across species, temperature (cool or warm), and the species × temperature interaction. (PDF)

S5 Table Analysis of variance of a linear model testing for differences in leaf shape in *Acer negundo* and *Carpinus caroliniana* across life stage (seedling or sapling), temperature (cool or warm), and the life stage × temperature interaction. (PDF)

S1 Dataset All leaf physiognomic data. (XLSX)

## References

[1] Bailey IW, Sinnott EW. A botanical index of Cretaceous and Tertiary climates. Science. 1915;41: 831–834. 10.1126/science.41.1066.831 17835989

[2] Bailey IW, Sinnott EW. The climatic distribution of certain types of angiosperm leaves. American Journal of Botany. 1916;3: 24–39.

[3] Wolfe JA. Temperature parameters of humid to mesic forests of eastern Asia and relation to forests of other regions of the Northern Hemisphere. U.S. Geological Survey Professional Paper. 1979;1106: 1–37.

[4] Greenwood DR, Wilf P, Wing SL, Christophel DC. Paleotemperature estimation using leaf-margin analysis: is Australia different?. Palaios. 2004;19: 129–142.

[5] Li Y, Wang Z, Xu X, Han W, Wang Q, Zou D. Leaf margin analysis of Chinese woody plants and the constraints on its application to palaeoclimatic reconstruction. Global Ecology and Biogeography. 2016;25: 1401–1415.

[6] Peppe DJ, Royer DL, Cariglino B, Oliver SY, Newman S, Leight E, et al Sensitivity of leaf size and shape to climate: global patterns and paleoclimatic applications. New Phytologist. 2011;190: 724–739. 10.1111/j.1469-8137.2010.03615.x 21294735

[7] Yang J, Spicer RA, Spicer TE, Arens NC, Jacques FM, Su T, et al Leaf form–climate relationships on the global stage: an ensemble of characters. Global Ecology and Biogeography. 2015;24: 1113–1125.

[8] Wolfe JA. A method of obtaining climatic parameters from leaf assemblages. U.S. Geological Survey Bulletin. 1993;2040: 1–171.

[9] Wilf P. When are leaves good thermometers? A new case for leaf margin analysis. Paleobiology. 1997;23: 373–390.

[10] Li SF, Jacques FM, Spicer RA, Su T, Spicer TE, Yang J, et al Artificial neural networks reveal a high-resolution climatic signal in leaf physiognomy. Palaeogeography, Palaeoclimatology, Palaeoecology. 2016;442: 1–11.

[11] Royer DL, Wilf P. Why do toothed leaves correlate with cold climates? Gas exchange at leaf margins provides new insights into a classic paleotemperature proxy. International Journal of Plant Sciences. 2006;167: 11–18.

[12] Feild TS, Sage TL, Czerniak C, Iles WJ. Hydathodal leaf teeth of Chloranthus japonicus (Chloranthaceae) prevent guttation‐induced flooding of the mesophyll. Plant, Cell & Environment. 2005;28:1179–1190.

[13] Givnish TJ, Kriebel R. Causes of ecological gradients in leaf margin entirety: Evaluating the roles of biomechanics, hydraulics, vein geometry, and bud packing. American Journal of Botany. 2017;104: 354–366. 10.3732/ajb.1600287 28232316

[14] Edwards EJ, Spriggs EL, Chatelet DS, Donoghue MJ. Unpacking a century‐old mystery: Winter buds and the latitudinal gradient in leaf form. American Journal of Botany. 2016;103: 975–978. 10.3732/ajb.1600129 27221280

[15] Royer DL. Leaf shape responds to temperature but not CO2 in Acer rubrum. PloS ONE. 2012;7: e49559 10.1371/journal.pone.0049559 23152921 PMC3495865

[16] Chitwood DH, Rundell SM, Li DY, Woodford QL, Yu TT, Lopez JR, et al Climate and developmental plasticity: interannual variability in grapevine leaf morphology. Plant Physiology. 2016;170: 1480–1491. 10.1104/pp.15.01825 26826220 PMC4775139

[17] Royer DL, McElwain JC, Adams JM, Wilf P. Sensitivity of leaf size and shape to climate within *Acer rubrum* and *Quercus kelloggii*. New Phytologist. 2008;179: 808–817. 10.1111/j.1469-8137.2008.02496.x 18507771

[18] Royer DL, Meyerson LA, Robertson KM, Adams JM. Phenotypic plasticity of leaf shape along a temperature gradient in Acer rubrum. PLoS ONE. 2009;4: e7653 10.1371/journal.pone.0007653 19893620 PMC2764093

[19] Royer DL, Wilf P, Janesko DA, Kowalski EA, Dilcher DL. Correlations of climate and plant ecology to leaf size and shape: potential proxies for the fossil record. American Journal of Botany. 2005;92: 1141–1151. 10.3732/ajb.92.7.1141 21646136

[20] Hinojosa LF, Pérez F, Gaxiola A, Sandoval I. Historical and phylogenetic constraints on the incidence of entire leaf margins: insights from a new South American model. Global Ecology and Biogeography. 2011;20: 380–390.

[21] Glade-Vargas N, Hinojosa LF, Leppe M. Evolution of climatic related leaf traits in the family Nothofagaceae. Frontiers in Plant Science. 2018;9 10.3389/fpls.2018.01073 30100913 PMC6073098

[22] Little SA, Kembel SW, Wilf P. Paleotemperature proxies from leaf fossils reinterpreted in light of evolutionary history. PLoS ONE. 2010;5: e15161 10.1371/journal.pone.0015161 21203554 PMC3008682

[23] Huff PM, Wilf P, Azumah EJ. Digital future for paleoclimate estimation from fossil leaves? Preliminary results. Palaios. 2003;18: 266–274.

[24] R Development Core Team. 2018. A language and environment for statistical computing.v3.5.1. R Foundation for Statistical Computing, Vienna, Austria.

[25] Lenth R, Love J, Hervé M. 2017 Package ‘emmeans’. American Statistician. 2010;34: 216–221.

