# Supplementary material for: Experimental evidence for species-dependent responses in leaf shape to temperature: Implications for paleoclimate inference
Source: PLoS One. 2019 Jun 21;14(6):e0218884. doi: 10.1371/journal.pone.0218884 (PMC6588257; doi:10.1371/journal.pone.0218884)
Supplement: S1 Text — Methods for cultivating plants in the growth cabinet experiment and for fractal dimension measurements. (PDF) [file pone.0218884.s001.pdf]

Methods for cultivating plants in the growth cabinet experiment

Seeds of *A. negundo* were purchased from TreeSeeds.com (Winooski, Vermont, USA) and grown in 10.5 cm by 12 cm deep square pots. Seeds of *B. lenta* were purchased from TreeHelp.com (Buffalo, New York, USA) and grown in 7.5 cm by 9.5 cm deep square pots. Seeds of *C. caroliniana* were purchased from Sheffield's Seed Co., Inc. (Locke, New York, USA) and grown in 7.5 cm by 9.5 cm deep square pots. Seeds of *Q. rubra* were collected from a single tree on Wesleyan University's campus (Middletown, Connecticut, USA) and grown in 11 cm by 20 cm deep square pots. All seeds were first soaked in water for 24 hours and then cold stratified for 90 (*A. negundo*, *C. caroliniana*) or 60 days (*B. lenta*, *Q. rubra*).

Bare-root, one-foot tall saplings of *A. negundo* were purchased from Wholesale Nursery Co. (Altamont, Tennessee, USA) and grown in 10.5 cm by 12 cm deep square pots. Bare root, one-foot tall saplings of *C. caroliniana* were purchased from North American Habitat (Free Soil, Michigan, USA) and grown in 15 cm diameter by 14 cm deep round pots. Bare root 4 inch tall seedlings of *I. opaca* were purchased from Mountain Seedlings (Seattle, Washington, USA) and grown in 10.5 cm by 12 cm deep square pots. One-foot tall saplings of *O. virginiana* were purchased from White Oak Nursery (Geneva, New York, USA) and grown in 11 cm by 20 cm deep square pots.

Methods for fractal dimension measurements

We computed the fractal dimension of each leaf following a procedure from Western Carolina University (http://paws.wcu.edu/emcnelis/SV/UsingImageJ.pdf). After the measurement scale is set in ImageJ, the image is then converted to grayscale. The image is

converted to binary following these menu options: Process → Binary → Make Binary. The leaf in the image is then selected using the rectangular selection tool. Using the following menu options will bring up a pop-up box with more options: Analyze → Tools → Fractal Box Count. By selecting OK, this will create a graph of the line of best fit for the log(count) vs. log(box size) data as well as a table of results and the fractal dimension itself.
