# Supplementary material for: Experimental evidence for species-dependent responses in leaf shape to temperature: Implications for paleoclimate inference
Source: PLoS One. 2019 Jun 21;14(6):e0218884. doi: 10.1371/journal.pone.0218884 (PMC6588257; doi:10.1371/journal.pone.0218884)
Supplement: S2 Table — (PDF) [file pone.0218884.s003.pdf]

S2 Table. Linear model results for saplings testing for the effect of temperature on leaf shape within species.

	Acer negundo			*Carpinus caroliniana*			*Ostrya virginiana*		
**Variable**	**Warm treatment**	**Cool treatment**	***P***	**Warm treatment**	**Cool treatment**	***P***	**Warm treatment**	**Cool treatment**	***P***
**Tooth abundance**									
Number of teeth	1.74 (0.36)	3.17 (0.19)	1.00	128.51 (11.12)	179.91 (4.58)	**<0.001**	103.24 (8.39)	107.00 (8.81)	1.00
Number of teeth / internal perimeter (cm^{-1})	0.13 (0.02)	0.23 (0.04)	1.00	9.62 (0.81)	14.05 (0.82)	**<0.001**	5.79 (0.57)	6.97 (0.93)	0.84
Number of teeth / blade area (cm^{-2})	0.51 (0.09)	0.67 (0.13)	1.00	14.26 (1.77)	23.23 (2.63)	**0.005**	6.79 (0.88)	10.20 (2.67)	0.80
**Tooth size**									
Tooth area (cm^2)	0.05 (0.02)	0.13 (0.03)	0.83	0.22 (0.02)	0.20 (0.03)	1.00	0.54 (0.09)	0.56 (0.10)	1.00
Average tooth area (cm^2)	0.02 (0.01)	0.03 (0.01)	**0.02**	0.002 (0.0004)	0.001 (0.0002)	1.00	0.01 (0.001)	0.01 (0.001)	1.00
Tooth area / internal perimeter (cm)	0.003 (0.001)	0.009 (0.002)	0.46	0.02 (0.002)	0.01 (0.002)	0.94	0.03 (0.003)	0.03 (0.005)	0.64
Tooth area / blade area	0.01 (0.003)	0.02 (0.004)	0.21	0.02 (0.003)	0.02 (0.002)	0.98	0.03 (0.002)	0.05 (0.01)	0.22
**Leaf dissection**									
Circularity	0.27 (0.02)	0.29 (0.02)	0.99	0.42 (0.01)	0.40 (0.01)	0.90	0.27 (0.02)	0.24 (0.02)	0.84
Perimeter ratio	1.02 (0.004)	1.05 (0.01)	0.98	1.27 (0.02)	1.27 (0.02)	1.00	1.53 (0.05)	1.61 (0.07)	0.50
Feret diameter ratio	0.50 (0.01)	0.57 (0.01)	**<0.001**	0.70 (0.01)	0.69 (0.01)	0.97	0.65 (0.01)	0.64 (0.01)	1.00
Fractal dimension	1.80 (0.02)	1.83 (0.01)	0.49	1.87 (0.005)	1.86 (0.005)	0.99	1.89 (0.005)	1.88 (0.01)	0.99

See Table 1 in main text for definitions of leaf shape variables. Values in parentheses are the standard error of the mean. *P* is the probability that there is no difference in leaf shape within a species between the temperature treatments; values in bold are <0.05. *P*-values are based on estimated marginal means (EMMs; see Methods).
