# Supplementary material for: Experimental evidence for species-dependent responses in leaf shape to temperature: Implications for paleoclimate inference
Source: PLoS One. 2019 Jun 21;14(6):e0218884. doi: 10.1371/journal.pone.0218884 (PMC6588257; doi:10.1371/journal.pone.0218884)
Supplement: S3 Table — (PDF) [file pone.0218884.s004.pdf]

S3 Table. Analysis of variance of a linear model testing for differences in leaf shape in seedlings across species, temperature (cool or warm), and the species × temperature interaction.

Variable	Effect	df	SS	F	*P*
**Tooth abundance**					
Number of teeth	Species	3	34003	197	**<0.001**
	Temperature	1	636	11.0	**0.002**
	Interaction	3	3406	19.7	**<0.001**
	Residuals	54	3109		
Number of teeth / internal perimeter	Species	3	149	199	**<0.001**
	Temperature	1	5.73	23.0	**<0.001**
	Interaction	3	6.19	8.27	**<0.001**
	Residuals	54	13.5		
Number of teeth / blade area	Species	3	284	64.2	**<0.001**
	Temperature	1	16.5	11.2	**0.002**
	Interaction	3	6.79	1.53	0.22
	Residuals	54	79.9		
**Tooth size**					
Tooth area	Species	2	2.43	5.24	**0.009**
	Temperature	1	0.785	3.39	0.07
	Interaction	2	1.20	2.59	0.09
	Residuals	42	9.73		
Average tooth area	Species	2	0.0237	11.4	**<0.001**
	Temperature	1	0.00226	2.17	0.15
	Interaction	2	0.00276	1.33	0.28
	Residuals	42	0.0436		
Tooth area / internal perimeter	Species	2	0.00455	10.1	**<0.001**
	Temperature	1	0.00217	9.66	**0.003**
	Interaction	2	0.00217	4.82	**0.01**
	Residuals	42	0.00944		
Tooth area / blade area	Species	2	0.00816	23.7	**<0.001**
	Temperature	1	0.00377	21.9	**<0.001**
	Interaction	2	0.00671	19.5	**<0.001**
	Residuals	42	0.00721		
**Leaf dissection**					
Circularity	Species	3	0.666	95.5	**<0.001**
	Temperature	1	0.0878	37.8	**<0.001**
	Interaction	3	0.0478	6.87	**<0.001**
	Residuals	54	0.125		
Perimeter ratio	Species	3	2.34	151	**<0.001**
	Temperature	1	0.0666	12.9	**<0.001**
	Interaction	3	0.00944	0.609	0.61
	Residuals	54	0.279		
Feret diameter ratio	Species	3	0.153	74.1	**<0.001**
	Temperature	1	0.000961	1.40	0.24
	Interaction	3	0.00654	3.17	**0.03**
	Residuals	54	0.0371		
Fractal dimension	Species	3	0.0187	14.1	**<0.001**
	Temperature	1	0.000006	0.0144	0.91
	Interaction	3	0.00316	2.39	0.08
	Residuals	54	0.0238		

See Table 1 in main text for definitions of leaf shape variables. df = degrees of freedom; SS = sum of squares; F = F statistic; *P* = probability that there is no difference in leaf shape due to the tested factor. *P*-values in bold are <0.05.
