# Supplementary material for: Experimental evidence for species-dependent responses in leaf shape to temperature: Implications for paleoclimate inference
Source: PLoS One. 2019 Jun 21;14(6):e0218884. doi: 10.1371/journal.pone.0218884 (PMC6588257; doi:10.1371/journal.pone.0218884)
Supplement: S4 Table — (PDF) [file pone.0218884.s005.pdf]

S4 Table. Analysis of variance of a linear model testing for differences in leaf shape in saplings across species, temperature (cool or warm), and the species × temperature interaction.

Variable	Effect	df	SS	F	*P*
**Tooth abundance**					
Number of teeth	Species	2	166498	295	**<0.001**
	Temperature	1	3974	14.1	**<0.001**
	Interaction	2	5315	9.41	**<0.001**
	Residuals	32	9036		
Number of teeth / internal perimeter	Species	2	953	191	**<0.001**
	Temperature	1	37.2	14.9	**<0.001**
	Interaction	2	35.0	7.02	**0.003**
	Residuals	32	79.9		
Number of teeth / blade area	Species	2	2318	63.7	**<0.001**
	Temperature	1	172	9.48	**0.005**
	Interaction	2	138	3.80	**0.03**
	Residuals	32	582		
**Tooth size**					
Tooth area	Species	2	1.27	41.9	**<0.001**
	Temperature	1	0.00582	0.385	0.54
	Interaction	2	0.0196	0.649	0.53
	Residuals	32	0.484		
Average tooth area	Species	2	0.00502	30.7	**<0.001**
	Temperature	1	0.000311	3.81	0.06
	Interaction	2	0.000593	3.63	**0.04**
	Residuals	32	0.00261		
Tooth area / internal perimeter	Species	2	0.00386	62.4	**<0.001**
	Temperature	1	0.0000565	1.83	0.19
	Interaction	2	0.000145	2.34	0.11
	Residuals	32	0.000990		
Tooth area / blade area	Species	2	0.00364	21.8	**<0.001**
	Temperature	1	0.000399	4.78	**0.04**
	Interaction	2	0.000533	3.19	0.05
	Residuals	32	0.00267		
**Leaf dissection**					
Circularity	Species	2	0.172	55.4	**<0.001**
	Temperature	1	0.00115	0.740	0.40
	Interaction	2	0.00329	1.06	0.36
	Residuals	32	0.0497		
Perimeter ratio	Species	2	1.71	172	**<0.001**
	Temperature	1	0.00784	1.58	0.22
	Interaction	2	0.0106	1.06	0.36
	Residuals	32	0.159		
Feret diameter ratio	Species	2	0.192	147	**<0.001**
	Temperature	1	0.00389	5.95	**0.02**
	Interaction	2	0.0131	10.1	**<0.001**
	Residuals	32	0.0209		
Fractal dimension	Species	2	0.0307	18.6	**<0.001**
	Temperature	1	0.000127	0.153	0.70
	Interaction	2	0.00311	1.88	0.17
	Residuals	32	0.0264		

See Table 1 in main text for definitions of leaf shape variables. df = degrees of freedom; SS = sum of squares; F = F statistic; *P* = probability that there is no difference in leaf shape due to the tested factor. *P*-values in bold are <0.05.
