# Supplementary material for: Experimental evidence for species-dependent responses in leaf shape to temperature: Implications for paleoclimate inference
Source: PLoS One. 2019 Jun 21;14(6):e0218884. doi: 10.1371/journal.pone.0218884 (PMC6588257; doi:10.1371/journal.pone.0218884)
Supplement: S5 Table — (PDF) [file pone.0218884.s006.pdf]

S5 Table. Analysis of variance of a linear model testing for differences in leaf shape in *Acer negundo* and *Carpinus caroliniana* across life stage (seedling or sapling), temperature (cool or warm), and the life stage × temperature interaction.

Variable	Effect	df	SS	F	*P*
**Tooth abundance**					
Number of teeth	Life stage	1	29975	8.42	**0.005**
	Temperature	1	7314	2.05	0.16
	Interaction	1	177	0.0497	0.82
	Residuals	52			
Number of teeth / internal perimeter	Life stage	1	198	9.26	**0.004**
	Temperature	1	40.0	1.87	0.18
	Interaction	1	4.60	0.215	0.64
	Residuals	52	1114		
Number of teeth / blade area	Life stage	1	583	9.98	**0.003**
	Temperature	1	116	1.99	0.16
	Interaction	1	39.6	0.678	0.41
	Residuals	52	3040		
**Tooth size**					
Tooth area	Life stage	1	6.24	52.4	**<0.001**
	Temperature	1	0.625	5.26	**0.03**
	Interaction	1	0.480	4.04	**0.05**
	Residuals	52	6.19		
Average tooth area	Life stage	1	0.0124	11.9	**0.001**
	Temperature	1	0.00132	1.27	0.26
	Interaction	1	0.000061	0.0585	0.81
	Residuals	52	0.0540		
Tooth area / internal perimeter	Life stage	1	0.0206	119	**<0.001**
	Temperature	1	0.000749	4.31	**0.04**
	Interaction	1	0.000493	2.84	0.10
	Residuals	52	0.00904		
Tooth area / blade area	Life stage	1	0.0269	94.6	**<0.001**
	Temperature	1	0.000667	2.34	0.13
	Interaction	1	0.000114	0.401	0.53
	Residuals	52	0.0148		
**Leaf dissection**					
Circularity	Life stage	1	0.131	16.0	**<0.001**
	Temperature	1	0.041	4.94	**0.03**
	Interaction	1	0.034	4.14	**0.05**
	Residuals	52	0.427		
Perimeter ratio	Life stage	1	0.156	6.37	**0.01**
	Temperature	1	0.035	1.43	0.24
	Interaction	1	0.216	0.882	0.35
	Residuals	52	1.272		
Feret diameter ratio	Life stage	1	0.215	46.7	**<0.001**
	Temperature	1	0.00285	0.618	0.44
	Interaction	1	0.00309	0.670	0.42
	Residuals	52	0.240		
Fractal dimension	Life stage	1	0.011	9.13	**0.004**
	Temperature	1	0.000799	0.641	0.42
	Interaction	1	0.000019	0.0153	0.90
	Residuals	52	0.0649		

See Table 1 in main text for definitions of leaf shape variables. df = degrees of freedom; SS = sum of squares; F = F statistic; *P* = probability that there is no difference in leaf shape due to the tested factor. *P*-values in bold are <0.05.
